# Metabolomic Profile Modification in the Cerebellum of Mice Repeatedly Exposed to Khat and Treated with β-Lactamase Inhibitor, Clavulanic Acid

**DOI:** 10.3390/metabo14120726

**Published:** 2024-12-23

**Authors:** Abdulkareem A. Alanezi

**Affiliations:** Department of Pharmaceutics, College of Pharmacy, University of Hafr Al Batin, Hafr Al Batin 39524, Saudi Arabia; aalanezi@uhb.edu.sa

**Keywords:** khat, metabolomic, cerebellum, clavulanic acid, GC-MS, gluconeogenesis

## Abstract

Background/Objectives: Catha edulis, commonly known as khat, is used for its psychoactive effects and is considered a natural amphetamine. The current study investigated the metabolomic profile in the cerebellum of mice after repeated exposure to khat and evaluated the effects of clavulanic acid on the metabolomic profile in the cerebellum in khat-treated mice. Methods: Male C67BL/6 mice that were 6–9 weeks old were recruited and divided into three groups: the control group was treated with 0.9% normal saline for 17 days; the khat group was given khat extract at a dose of 360 mg/kg via the intraperitoneal (i.p) route for 17 days; and another khat group was treated with khat for 17 days and clavulanic acid at a dose of 5 mg/kg for the last 7 days (days 11–17). At the end of the 17th day, the animals were sacrificed, and their brains were immediately collected and stored at −80 °C. The cerebellum region of the brain was isolated in each group by micropuncture using cryostat and underwent a metabolomics study via Gas Chromatography/Mass Spectroscopy (GC/MS). The total peak area ratios of the selected metabolites in the cerebellum after repeated exposure to the khat extract were significantly reduced (*p* < 0.05) and treatment of the khat group with clavulanic acid significantly increased (all *p* < 0.05) the total peak areas ratios of the selected metabolites when compared to their corresponding areas in the alternative khat group. These levels of selected metabolites were further confirmed by observing the metabolite peak area ratios and performing a heat map analysis and a principal compartment analysis of the samples in the cerebellum. Results: A network analysis of altered metabolites in the cerebellum showed a strong correlation between the different metabolites, which showed that an increase in one metabolite can modulate the levels of others. An analysis using the MetaboAnalyst software revealed the involvement of selected altered metabolites like lactic acid in many signaling pathways, like gluconeogenesis, while enrichment analysis data showed altered pathways for pyruvate metabolism and disease pathogenesis. Finally, a network analysis showed that selected metabolites were linked with other metabolites, indicating drug–drug interactions. Conclusions: The present study showed that repeated exposure of mice to khat altered the levels of various metabolites in the cerebellum which are involved in the pathogenesis of different diseases, signaling pathways, and interactions with the pharmacokinetic profile of other therapeutic drugs. The treatment of khat-treated mice with clavulanic acid positively modified the metabolomics profile in the cerebellum and increased the levels of the altered metabolites.

## 1. Introduction

Substance use disorders result in psychological and physiological impairments that occur in the human body due to repeated exposure to a psychoactive agent present in a drug or food. Drug abuse results from multiple factors which include behavioral, cognitive, environmental, genetic, epigenetic, motivational, and emotional mechanisms, which involve the responses of the brain to reward and anxiety [1]. The mesolimbic system, which is involved in physiological and cognitive reward in the brain, is related to many reward-related activities like feeding, exercise, sex, substance use, and social interactions which lead to elevated levels of dopamine [2]. The magnitude of an addiction is determined by the brain structure that is responsible for reward pathways, i.e., the ventral tegmental area, ventral striatum and nucleus accumbens (NAc), amygdala, or the hippocampus, among other areas of the brain [3]. Drugs of abuse stimulate the reward system by activating the mesolimbic system and the release of dopamine from the target nuclei [2]. This release of dopamine is increased in the NAc brain area. Among the many altered mechanisms is the upregulation or downregulation of receptors and transporters of neurotransmitters such as glutamate. Glutamate receptor neuroadaptation is one of the mechanisms which is triggered by continuous exposure to drugs of abuse [4]. Glutamate is an excitatory neurotransmitter which is involved in various functions like learning and memory, and therefore, should be kept at a normal physiological concentration in the brain, as higher than normal concentrations lead to excitotoxicity, which is one of the factors of relapse in drug abuse [5].

A naturally occurring herb, *Catha edulis*, locally known as khat, is smuggled from the Yemeni border of Saudi Arabia and the Jazan district [6,7] and abused for its psychoactive effects [8,9], leading it to be labeled as a natural amphetamine [10] which is used for its effects including increased alertness, high energy, improved motivation, and hunger suppression [11]. Although chewing khat is legal in a few countries, it is still banned in Saudi Arabia, Malaysia, and the USA [12]. The overall prevalence of khat chewing in the studied population was 21.4% (15.2% in colleges versus 21.5% in schools) in the Jazan district of Saudi Arabia [13], with reports showing a total of 3.8% female khat chewers and 37.70% male khat chewers. It has been reported to be involved in 77% of fatalities and crimes in the Jazan district [14]. A phytochemical analysis of khat revealed the presence of cathinone, which is the main psychoactive alkaloid, while cathine is a less potent psychoactive alkaloid component [15] which affects both the central as well as peripheral nervous system. Cathinone is the most abundant alkaloid [16], while cathine is present in lower concentrations in the fresh leaves of *Catha edulis.* However, upon drying, cathinone is converted to cathine, which subsequently turns into norephedrine [17]. Chronic administration of synthetic cathinone increases the levels of norepinephrine and glutamate in different areas of the brain, but minimal changes in dopamine levels were observed in female rats [18]. Increased levels of glutamate and norepinephrine in multiple parts of the brain indicate that excitotoxicity can be a potential therapeutic target for khat use disorder.

Studies show that the uptake of glutamate is regulated by astrocytes from excitatory amino acid transporters (EAATs) [19]. This excitatory neurotransmitter is transported into cells by EAATs, along with sodium, against the concentration gradient. The removal of glutamate in normal physiological circumstances is favored by potassium efflux along with hydrogen influx [20]. There are two types of EAATs which are involved in the regulation of glutamate in the brain: Glutamate-aspartate transporter (GLAST), which is also called excitatory amino acid transporter 1(EAAT)1, is expressed in the cerebellum, while glutamate transporter 1, GLT-1, which is also called excitatory amino acid transporter 2 (EAAT 2), is a transporter of glutamate in the forebrain [19]. However, the latter is also expressed in the cerebellum. This glutamate excitotoxicity is associated with many neurodegenerative diseases like Alzheimer’s disease, Parkinson’s disease [21], amyotrophic lateral sclerosis, and Huntington’s disease [22]. Any impairment in this glutamate–EAAT pathway might lead to a higher concentration of glutamate, which leads to cell injury and glutamate-induced excitotoxicity [19]. In preclinical models of drugs of abuse such as amphetamine, hydrocodone, cocaine, and nicotine, the glutamate–EAAT pathway is impaired by decreased expression of GLT-1 in the NAc [23,24], which could ultimately increase the extracellular levels of glutamate [25] and consequently result in decreased clearance of glutamate [26].

Decreased expression of the GLT-1 protein has been targeted in many studies to attenuate the drug seeking behavior in the mesocorticolimbic brain regions by using ceftriaxone and β-lactamase inhibitor [5,23,27]. The β-lactamase inhibitor, clavulanic acid, has been reported to upregulate the GLT-1 protein in NAc and attenuate drug seeking behavior for ethanol and amphetamine [28,29]. Recently reported data utilized clavulanic acid in mice which were repeatedly exposed to khat extract and observed an attenuation of neurobehavioral changes in the form of improvements in memory and anxiety disorders by upregulation of GLT-1 protein in NAc [30]. To the best of our knowledge, none of the studies to date has reported the impact of khat on the metabolomic profile in the cerebellum brain region or the impact of clavulanic acid treatments on the metabolomic profile after khat exposure. The current study was performed to investigate the metabolomics profile in the cerebellum brain area in mice after repeated exposure to khat. Secondly, our study also set out to evaluate the effects of clavulanic acid on the metabolomic profile in the cerebellum in khat treated mice.

## 2. Materials and Methods

### 2.1. Chemicals

Medicinal plant khat extract was prepared by following a method previously reported [30,31]. Briefly, one kilogram (1 kg) of plant material was washed, soaked, and macerated in volume of 2.5 L of 80% aqueous ethanolic solution for 3 days (72 h) at room temperature with a spontaneous orbital shaker (Gallen Kamp, Cambridge, UK). After 72 h of continuous shaking, the supernatant layer was isolated using a filter paper with 0.45 μm size. The filtered extract was allowed to dry at room temperature and was later weighed to get a percentage yield. The dried filtered extract was placed in amber colored bottle and kept in the refrigerator at 4 °C. Other chemicals used in the laboratory for the experiment were prepared on daily basis. Clavulanic acid (Sigma-Aldrich, Darmstadt, Germany) was prepared fresh in 0.9% normal saline solution on daily basis to inject the laboratory animals.

### 2.2. Experimental Study

Male C67BL/6 mice, 6–9 weeks old and weighing 20–25 gms, were obtained for the study and housed in a transit animal house facility at the College of Pharmacy, King Saud University, Riyadh, Saudi Arabia, under a controlled environment with a 12 h day light cycle. The humidity in the transit animal room was kept between 40–60% while the temperature was maintained at 22 ± 2 °C. All mice had free access to standard rodent Chow food and water. Animals were acclimatized for 7 days before starting any procedure. All the procedures and protocols adopted to perform this study were approved by The Institutional Animal Care and Use Committee at King Saud University, Riyadh, Saudi Arabia, under the approval number (KSU-SE-22-53).

### 2.3. Study Design and Groups

**Figure 1:**
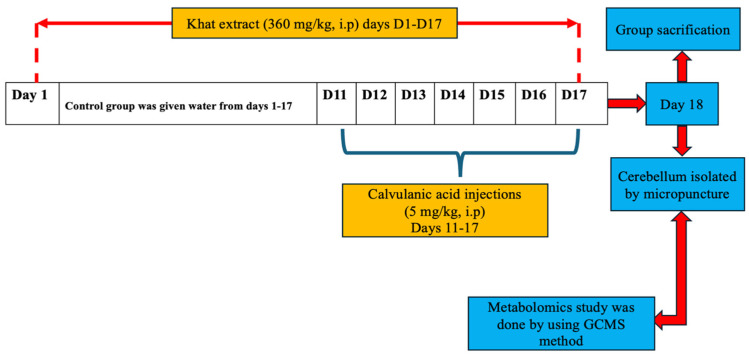
Schematic flow chart of the study protocol for different treatment groups.

The present study was set for 17 days of duration, as shown in the Figure 1 and reported previously [30]. All mice were divided into three groups (n = 4/group), which were given treatments as follow. Group 1: The control group was treated with 0.9% normal saline for 17 days. Group 2: The khat group was given khat extract at a dose of 360 mg/kg, intraperitoneal (i.p) route for 17 days [32]. Group 3: The khat/clavulanic acid group (administered khat 360 mg/kg, intraperitoneal route for 17 days) was treated with clavulanic acid at the dose of 5 mg/kg for the last seven days (days 11–17) [29].

### 2.4. Metabolomic Analysis

All groups were handled in such a way as to minimize the stress on the animals to avoid any physiological or biochemical changes in the brain. At the end of the 17th day, animals were sacrificed by cervical dislocation and brains were immediately collected and stored at −80 °C. The cerebellum region of the brain was isolated in each group by micropuncture using a cryostat, which was maintained at −20 °C using the mice atlas to indicate the cerebellum area. All cerebellum samples were then kept at −80 °C.

The stored cerebellum samples were further used for our metabolomics study using a procedure already reported [33]. The sample preparations were performed by ethanol derivatization, using GC grade ethanol [34]. Briefly, the cerebellum samples were defrosted under the control environment, homogenized with methanol to extract metabolites, and centrifuged at 10,000 rpm for 3 min at 4 °C. After centrifugation, a volume of 2 μL was taken from the supernatant layer and put in 2 mL vial of GC-MS. Samples were dried using liquid nitrogen. Samples were further treated with methoxamine hydrochloride (100 μL of 20 mg/mL) being added to the pyridine solution and put into incubation for 16 h after complete mixing with a vortex mixer. After completing the procedure of incubation, the samples were derivatized using BSTFA/TMCS (99/1, *v*/*v*) and 1 μL was injected using a split-mode injection device. A T mass spectrometer was used to determine the mass-to-charge ratio of each metabolite, Turbomass software Version 5.4 was used for data analysis, and a Clarus 600 gas chromatograph, connected to the GC-MS machine, was used for the operating procedures. Interpretations of the metabolomic data were performed using the MetaboAnalyst web-based platform. A set of methods like Heatmap analysis, score plotting, enrichment analysis, and sparse Partial Least Squares Discriminant Analysis (sPLS-DA) were used to further analyze significant differences and patterns of the obtained metabolomics data. Certain altered metabolites were chosen for enrichment analysis of the signaling pathways, pathogenesis, and to identify drug–drug interactions.

### 2.5. Statistical Analysis

The peak area of a single metabolite in each sample, including in the control group, was normalized to the average of peak areas of that metabolite in the control group, as performed previously [33]. One-way analysis of variance (ANOVA) was used to investigate the significant differences of each metabolite among the four study groups. An unpaired t test was further used to determine whether khat exposure could reduce the peak area ratios of these metabolites compared to control group and whether clavulanic acid could reverse these effects. Data are presented as mean ± SEM and statistical significance is considered when *p* < 0.05.

## 3. Results

### 3.1. Peak Areas of Total Metabolites in the Cerebellum of the Brain in Control, Khat, and Khat Treated with Clavulanic Acid Groups

**Figure 2:**
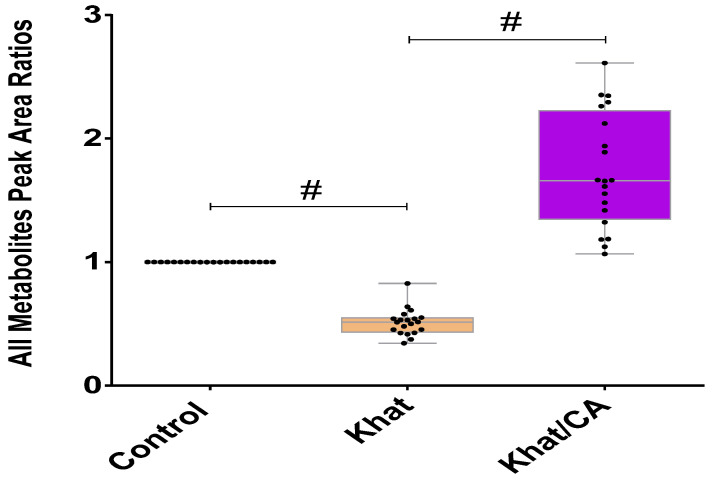
Peak area ratio of 20 metabolite means in the cerebellum after repeated exposure to khat extract and in the khat group treated with clavulanic acid. Data are presented as mean *±* SEM. Statistical significance is shown with #, which is *p* < 0.0001. Each dot represents one metabolite mean with total of 20 metabolite means.

The overall status of the concentrations of selected metabolites in the control, khat and khat treated with clavulanic acid groups is shown in Figure 2. One-way ANOVA of peak area ratios of 20 metabolites showed significant differences among the groups (*p* < 0.0001). An unpaired t test showed significant reductions in total mean metabolites (mean ± SEM) (*p* < 0.0001) in the cerebellum in mice after exposure to khat for 17 days, as compared to the control. Treatments of the khat group with clavulanic acid from day 11 through day 17 significantly increased the peak area ratios of all metabolite means in the cerebellum when compared to khat group (*p* < 0.0001) (Figure 2).

### 3.2. Peak Areas of Selected Metabolites in the Cerebellum in Control, Khat, and Khat-Clavulanic Acid Groups

**Figure 3:**
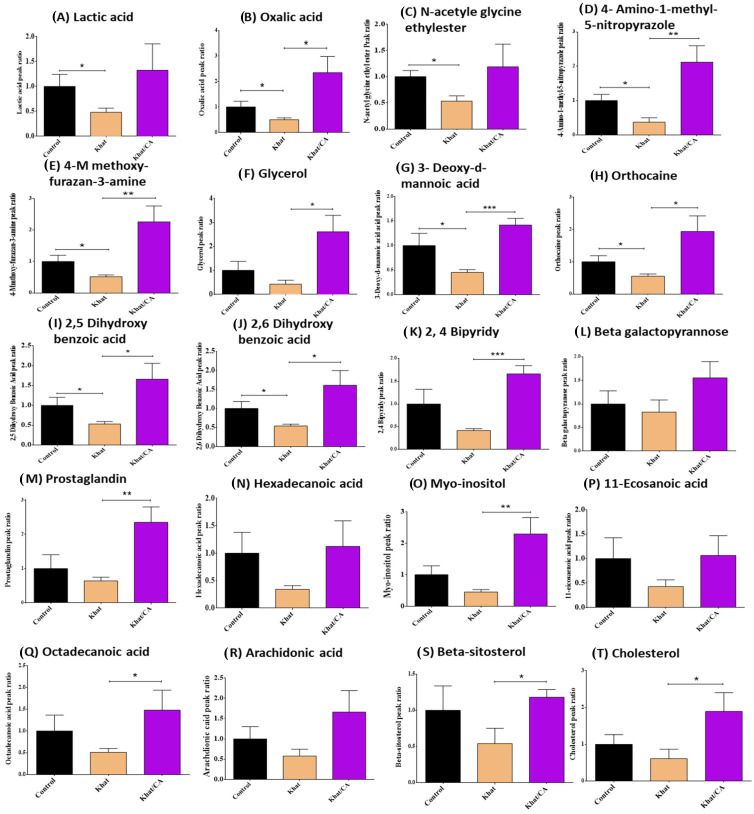
Peak area ratios of metabolites in the cerebellum in the control, khat, and khat-clavulanic acid groups. (**A**) Lactic acid. (**B**) Oxalic acid. (**C**) N-acetyle glycine ethylester. (**D**) 4-Amino-1-methyl-5-nitropyrazole. (**E**) 4-M methoxy-furazan-3-amine. (**F**) Glycerol. (**G**) 3-Deoxy-d-mannoic acid. (**H**) Orthocaine. (**I**) 2,5 Dihydroxy benzoic acid. (**J**) 2,6 Dihydroxy benzoic acid. (**K**) 2, 4 Bipyridy. (**L**) Beta galactopyrannose. (**M**) Prostaglandin. (**N**) Hexadecanoic acid. (**O**) Myo-inositol. (**P**) 11-Ecosanoic acid. (**Q**) Octadecanoic acid. (**R**) Arachidonic acid. (**S**) Beta-sitosterol. (**T**) Cholesterol. * *p* < 0.05; ** *p* < 0.01; *** *p* < 0.001.

Peak areas correspond to the concentrations of selected metabolites in the cerebellum brain area in the control, khat, and khat-clavulanic acid groups. One-way ANOVA did not show significant differences in lactic acid concentrations among groups (*p* > 0.05). However, a t test showed statistically reduced lactic acid in the khat group compared to the control group (*p* < 0.05) (Figure 3A). Our analysis showed statistically significant changes in oxalic acid among the groups (*p* < 0.05). Moreover, clavulanic acid treatment reversed (*p* < 0.05) khat-reduced oxalic acid (*p* < 0.05) (Figure 3B). Our statistical analysis did not show significant differences in n-acetyl glycine ethyl ester among the three groups (*p* > 0.05). However, a t test showed statistically significantly reduced n-acetyl glycine ethyl ester in the khat treated group compared to the control group (*p* < 0.05) (Figure 3C). Our analysis showed statistically significant changes in 4-amino-1-methyl-5-nitropyrazole among the three groups (*p* < 0.01). Moreover, clavulanic acid treatments reversed (*p* < 0.01) khat-reduced 4-amino-1-methyl-5-nitropyrazole (*p* < 0.05) (Figure 3D). Our analysis showed statistically significant changes in 4-methoxy-furazan-3-amine among the three groups (*p* < 0.01). Moreover, clavulanic acid treatments reversed (*p* < 0.01) khat-reduced 4-methoxy-furazan-3-amine (*p* < 0.05) (Figure 3E). A statistical analysis showed significant differences in glycerol among the three groups (*p* < 0.05). Moreover, a t test showed that the clavulanic acid-khat group had higher glycerol compared to the khat group (*p* < 0.05) (Figure 3F). Our analysis showed statistically significant changes in 3-deoxy-d-mannoic acid among the three groups (*p* < 0.01). Moreover, clavulanic acid treatment reversed (*p* < 0.01) khat-reduced 3-deoxy-D-mannoic acid (*p* < 0.05) (Figure 3G). Our analysis showed statistically significant changes in orthocaine among the three groups (*p* < 0.05). Moreover, clavulanic acid treatment reversed (*p* < 0.05) khat-reduced orthocaine (*p* < 0.05) (Figure 3H). Our analysis showed statistically significant changes in 2,5 dihydroxy benzoic acid among the three groups (*p* < 0.05). Moreover, clavulanic acid treatments reversed (*p* < 0.05) khat-reduced 2,5 dihydroxy benzoic acid (*p* < 0.05) (Figure 3I). Our analysis showed statistically significant changes in 2,6 dihydroxy benzoic acid among the three groups (*p* < 0.05). Moreover, clavulanic acid treatments reversed (*p* < 0.05) khat-reduced 2,6 dihydroxy benzoic acid (*p* < 0.05) (Figure 3J). Our statistical analysis showed significant differences in 2,4 bipyridyl among groups (*p* < 0.01). Moreover, a t test showed that the clavulanic acid-khat group had higher 2,4 bipyridyl compared to the khat group (*p* < 0.001) (Figure 3K). One-way ANOVA did not show significant differences in beta galactopyrannose among groups (*p* > 0.05) (Figure 3L). Our statistical analysis showed significant differences in prostaglandin among the three groups (*p* < 0.05). Moreover, a t test showed that the clavulanic acid-khat group had higher prostaglandin compared to the khat group (*p* < 0.01) (Figure 3M). One-way ANOVA did not show significant differences in hexadecenoic acid among groups (*p* > 0.05) (Figure 3N). Our statistical analysis showed significant differences in myo-inositol among the three groups (*p* < 0.05). However, a *t* test showed that the clavulanic acid-khat group had higher myo-inositol compared to the khat group (*p* < 0.01) (Figure 3O). Our statistical analysis did not show significant differences in 11-ecosanoic acid among the three groups (*p* > 0.05) (Figure 3P). Our statistical analysis did not show significant differences in octadecanoic acid among groups (*p* > 0.05). However, a *t* test showed statistically significantly increased octadecanoic acid in the khat-clavulanic acid group compared to the khat group (*p* < 0.05) (Figure 3Q). Our statistical analysis did not show significant differences in arachidonic acid among the three groups (*p* > 0.05) (Figure 3R). our the analysis did not show any significant differences in both beta-sitosterol and cholesterol among the groups, the khat-clavulanic acid group had higher beta-sitosterol (*p* < 0.05) and cholesterol (*p* < 0.05) compared to the khat group (Figure 3S,T).

### 3.3. Heatmap Analysis of Different Selected Metabolites in the Cerebellum in the Control, Khat, and Khat Treated with Clavulanic Acid Groups

A heat map analysis shows the expression levels of selected metabolites with different color schemes in different groups of the study. The control group showed average levels of all metabolites, while the khat group showed reduced levels of these metabolites (green color).

**Figure 4:**
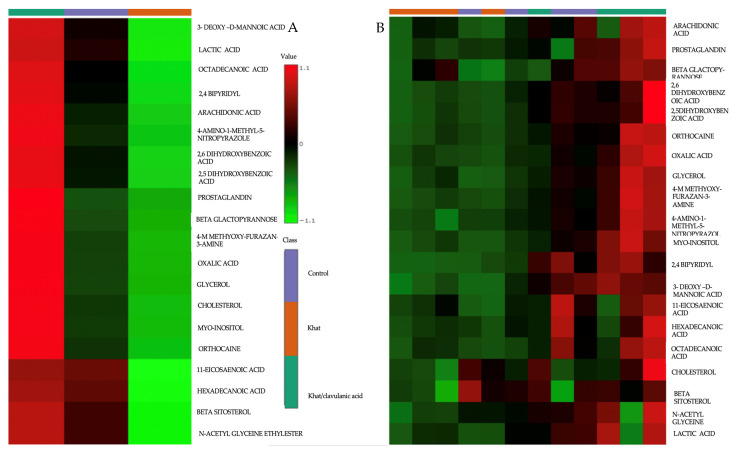
Heatmap analysis of detected metabolites in the cerebellum depicting (**A**) the average levels metabolite peak area ratios in the control, khat, and khat-clavulanic acid groups. (**B**) Sample metabolite peak area ratios in the control, khat, and khat-clavulanic acid groups. Experimental groups were classified as control (purple), khat (orange), and khat-clavulanic acid (green), and are shown in columns, while metabolites are shown in horizontal rows. The color scale to show the levels of these metabolite peak area ratios start from green (indicating minimum levels) to red (indicating maximum levels).

In addition, the khat group treated with clavulanic acid showed an observable increase in the levels of most of these metabolites, shown in red (Figure 4A). The data clearly indicate that the khat group, when treated with clavulanic acid, had reversed levels of metabolites. Moreover, within our sample evaluations, it was clearly that there were significant differences in the metabolomic profiles between the khat and control groups and between the khat and khat-clavulanic acid groups.

### 3.4. Principal Compartment Metabolomics Analysis in the Control, Khat, and Khat Treated with Clavulanic Acid Groups

**Figure 5:**
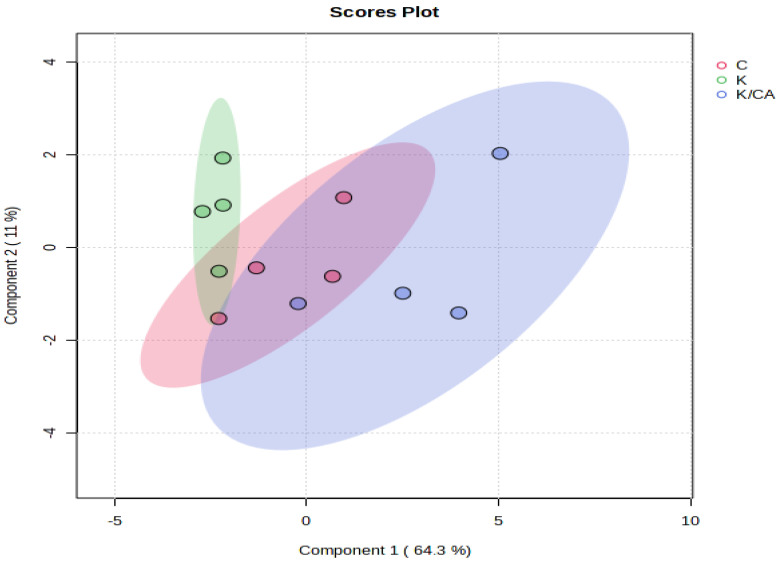
Sparse partial least squares discriminant analysis (sPLS-DA) of samples in the control, khat, and khat treated with clavulanic acid groups. A summary image of the segregation the metabolomic profiles in the cerebellum of the control (C) group (red), khat (K) (green), and khat group treated with clavulanic acid (K/CA) (purple).

A compartment analysis yielded a 3-D image of modifications in the metabolomic profile in the cerebellum in the control, khat, and khat treated with clavulanic acid. This analysis revealed the segregation of the metabolites between the samples obtained from control group and the khat untreated group, as well as clear segregation between the khat group and the khat-clavulanic acid group (Figure 5).

**Figure 6:**
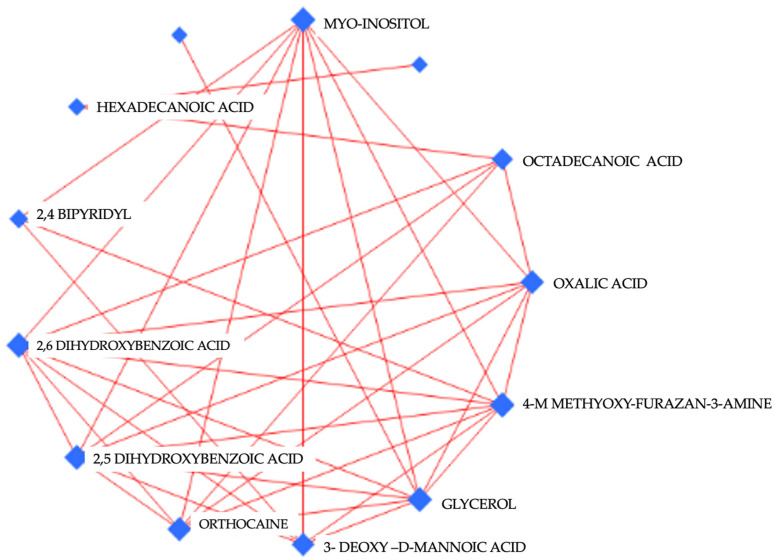
Network analysis of detected metabolites in the cerebellum of mice after repeated exposure to khat and treated with clavulanic acid.

A network analysis of altered metabolites in the cerebellum of the mice in the control, khat, and khat treated with clavulanic acid groups is shown in (Figure 6)**.** A network analysis indicated altered metabolites in the cerebellum part of the brain of the mice after repeated exposure to khat. This network analysis depicted the relationship and interactions among these altered metabolites in the khat exposure group. Each node indicated with blue color represents a metabolite, while the size of the blue nodes represents the magnitude of the change in the metabolite’s levels, i.e., the greater the size of the node, the greater the magnitude of the change in its levels. Red lines connecting the blue nodes show the associations of these metabolites with each other, indicating that if the level of one metabolite increased or decreased, there was a corresponding increase or decrease in the level of the second, connected metabolite

### 3.5. Enrichment Analysis of Selected Metabolites Altered in the Khat and Khat-Clavulanic Acid Groups

**Figure 7:**
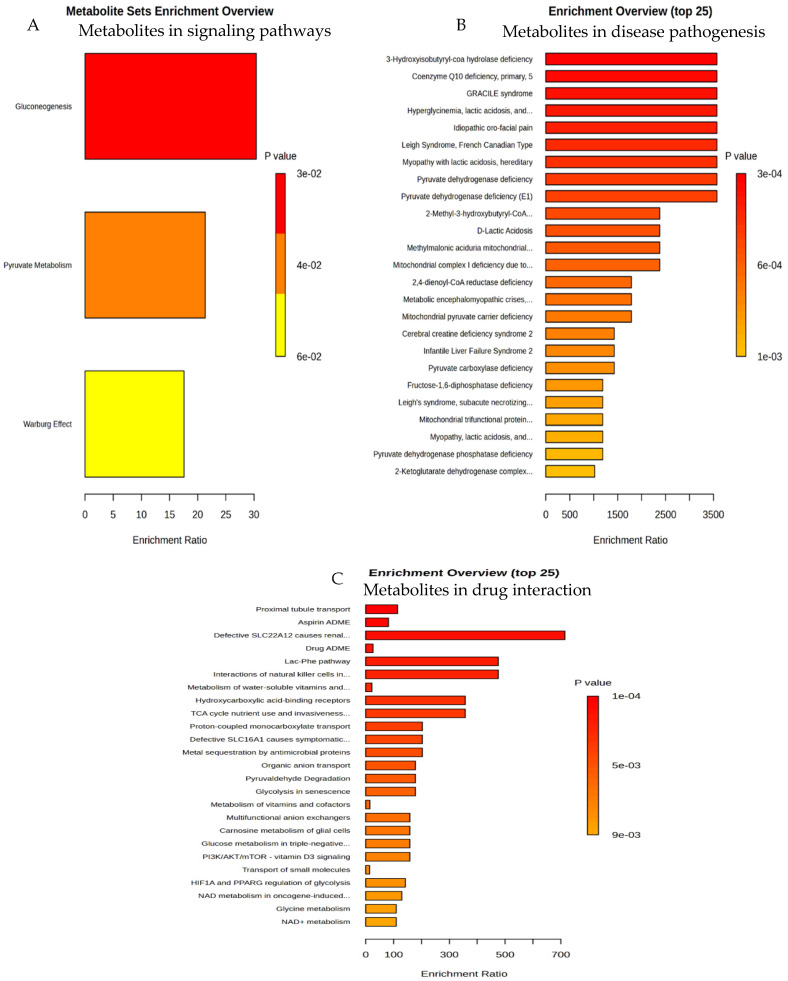
(**A**–**C**): Role of khat exposure in altering selected metabolites in signaling pathways (**A**), disease pathogenesis (**B**), and drug–drug interactions (**C**) using MetaboAnalyst software Version 6.

The obtained data of the selected metabolites were investigated using MetaboAnalyst software Version 6. The software generated enrichment analyses that revealed the involvement of these altered metabolites in many signaling pathways, such as gluconeogenesis, pyruvate metabolism (Figure 7A), disease pathogenesis (Figure 7B), and drug–drug interactions (Figure 7C).

Each bar in these three figures represents one activity as labelled, and the length of the bar represents the corresponding magnitude of the enrichment, as measured using an enrichment score, shown on the x-axis, while the y-axis represents different metabolic pathways, signaling pathways, and drug–drug interactions. The colors of the bars represent the intensity of the activity; Figure 7A indicates that gluconeogenesis was strongly modulated when the animals were repeatedly exposed to khat treatments.

## 4. Discussion

The current study was conducted to investigate the metabolomics profiles in the cerebellum area of the brain in mice after repeated exposure to khat. Secondly, the study was performed to evaluate the effects of clavulanic acid on these metabolomics profiles. The present study came up with two novel findings. Firstly, we found that repeated exposure to a khat extract altered the levels of various cerebellum metabolites which are involved in the pathogenesis of different diseases, signaling pathways, and interactions with the pharmacokinetic profiles of other therapeutic drugs. Secondly, clavulanic acid treatments in khat exposed mice positively modified the metabolomic profiles in the cerebellum and increased the levels of reduced metabolites.

The present study reported that repeated exposure to khat in mice decreased the peak areas of metabolites such as lactic acid, oxalic acid, 4-amino-1-methyl-5-nitropyrazole, 4-m methoxy-furazan-3-amine, 3-deoxy-d-mannoic acid, orthocaine, 2,5 dihydroxy benzoic acid, and 2, 6 dihydroxy benzoic acid in the cerebellum, compared to the control group. Alterations in the metabolomic profiles in the liver [33], kidney, lungs, and brain due to the use of psychoactive agents like fentanyl and synthetic 4-chloroethcathinone have been reported previously [35]. Likewise, in the present study, we observed that repeated exposure to drugs of abuse altered the metabolomic profile. The findings in the present study indicate that repeated exposure of khat alters metabolite levels in the cerebellum and can modulate the signaling pathways involved in different metabolisms, disease pathogeneses, and, to some extent, in the metabolism of various drugs [33]. It is important to know that 7-day treatment with clavulanic acid in a khat exposed group reversed the levels of all metabolites that were reduced in khat treated group, as indicated by assessing the peak areas of these metabolites. This suggests that all the altered metabolites and adverse effects associated with psychoactive drug use, including khat, may be reversed using clavulanic acid. It was expected that this would not only reduce drug-seeking behavior but would also modulate the various metabolic pathways which were altered following repeated khat exposure. Previously reported data showed that clavulanic acid treatment modified the glutamate pathway in the brain and can be a therapeutic option [36] to modify methamphetamine induced glutaminase mRNA changes in nucleus accumbens [37]. Although our finding is in line with previous data, we came up with the novel result that clavulanic acid treatment reverses the khat-induced modification of metabolites in the cerebellum.

A heatmap analysis of the metabolites detected in the cerebellum showed that khat treatment altered various metabolites, such as arachidonic acid, eicosanoid, prostaglandins, sterol, cholesterol, lactic acid, and oxalic acid, which are biomarkers of various signaling and metabolic pathways. These changes are similar to those observed in the liver after fentanyl overdose [38] or the administration of heroin [39]. Opiates are generally involved in the modulation of pain and inflammation [40]**,** while reduced levels of the metabolites of pain pathways indicated that khat treatment either reduced or modulated the them, i.e., either the arachidonic acid-prostaglandin [41] or arachidonic acid- eicosanoid pathway [42]. These metabolomic study findings are unique in the sense that khat treatment was found to modulate the pain pathway by altering the metabolomic profile in the cerebellum. Treatment with clavulanic acid to khat exposed group was found to reverse changes in the levels of these metabolites of pain and inflammation. These changes were further confirmed using principal compartment metabolomic analysis showing differences between the khat and control groups, with the clavulanic acid-khat group showing values approaching those of the control group. Keeping in view this modified metabolomic profile in the cerebellum, khat treatment is involved in the alteration of many other signaling pathways, such as the levels of sterol, cholesterol, lactic acid, and oxalic acid. A networking analysis indicated that these metabolites are related, meaning that increasing the levels of one metabolite will influence the levels of the others.

An analysis of selected metabolite levels in the cerebellum in the different samples showed that metabolites of lipid metabolism, such as cholesterol, arachidonic acid, and hexadecenoic acid, were decreased in the khat group, while treatment of the khat group with clavulanic acid reversed these changes. Similar findings were reported in a previous study, where lipid metabolism dysregulation data was observed following exposure to heroin, methamphetamine, and khat in other biological specimens, such as serum and urine [43,44,45,46]. Increased levels of lipid metabolism in the khat group treated with clavulanic acid revealed that lipid metabolite levels can be corrected or restored by clavulanic acid. Despite conflicting data reporting that khat chewing increases triglycerides levels in khat users when compared to non-khat users in Yemeni patients [46], in previous observations, Saudi khat users showed reductions in the levels of total cholesterol and low density lipoprotein [47]. This reducing effect of khat on the lipid profile was attributed to one of the phytoconstituents of khat, cathinone, which has amphetamine-like effects on lipid metabolism via modulating beta adrenergic receptors [48]. In the present study, the cerebellum metabolomic profiles of khat exposed mice showed reductions in the levels of cholesterol, which is consistent with the results of a prior study showing similar effects in human khat users [47]. Observations related to arachidonic acid in the present study were also in agreement with those of a previous study reporting decreased levels of arachidonic acid post fentanyl overdose and in schizophrenia [49,50]. Interestingly, the present study showed the novel modulatory effects of clavulanic acid on khat-induced impaired cerebellum metabolic profiles in mice.

A heatmap analysis of different samples of selected metabolite levels in the cerebellum showed that lactic acid was decreased in the khat group, and treatments with clavulanic acid reversed the lactic acid levels in mice exposed to khat. This indicates that many metabolic pathways that are linked with lactic acid can be modulated. A reduced lactate level is an indicator that energy depletion is greater than its supply to the body. This depletion of energy might be an indicator of mitochondrial dysfunction, which is an indicator of Parkinson disease [51] and cocaine abuse [52]. Lactate is produced from or converted to pyruvate by an enzyme, lactate dehydrogenate, in the cytosol; this process also involves nicotinamide adenine dinucleotide, which can then be utilized in glycolysis in the tricarboxylic acid cycle. The production of pyruvate from lactic acid is involved in glucose production in the liver, which is also called gluconeogenesis [53]. The present study revealed that lactic acid modifications in the cerebellum in the khat group were reversed by treatment with clavulanic acid.

Our heatmap analysis gives an insight into the modification in gluconeogenesis due to alterations in lactate-pyruvate metabolomic data in the khat group, supported by enrichment analysis data. Our enrichment analysis revealed the involvement of these altered metabolites in the cerebellum in many signaling pathways, such as gluconeogenesis, pyruvate metabolism, disease pathogenesis, and drug–drug interactions. This study reports novel findings, i.e., that three pathways, namely, gluconeogenesis, pyruvate metabolism, and Warburg effects, indicated the modulatory effects of the metabolic pathways in the cerebellum in a khat treated group of mice. Heatmap data revealed that the majority of the detected metabolites were altered in the khat group, including metabolites involved in the energy metabolism, which is consistent with previous studies on the thalamus and striatum showing similar findings after repeated exposure to cocaine [54]. Although the reported data for khat exposure on glucose were inconsistent, our enrichment analysis indicated that gluconeogenesis might be modulated by khat, which is in agreement with previous studies reporting that khat chewing raises blood glucose in normal individuals [55,56], and an association between khat chewing and non-insulin dependent diabetes mellitus has been established in data reported for humans [57]. The findings of the current study on lactate or the enrichment of gluconeogenesis pathways in the cerebellum may suggest that these effects of khat exposure can also occur in the systemic circulation, indicating that the gluconeogenesis pathway is modified in khat treated animals, while treatment with clavulanic acid can reverse these effects.

Our enrichment analysis results revealed that khat may interfere the pharmacokinetics of other drugs. This finding is in agreement with previously reported findings on an ethanolic extract of khat and cathinone, which showed strong interactions between cathinone and human cytochrome P450 (CYP) 2C9, CYP2D6, and CYP3A4 enzymes activities [58]. Khat has been reported to interact with many therapeutic agents that are frequently used in clinical practice, such as antibiotics [59] and anesthetic agents [60]. It was found that khat exposure inhibits the metabolic enzymes of cytochrome P450 (CYP450, CYP2D6 and CYP3A4 isoforms), which causes alterations in the pharmacokinetic profiles of drugs that act on central nervous system, such as sertraline, vilazodone, clomipramine, and aripiprazole, producing drug toxicity [61]. Many such type of interactions have been reported in previous studies [62]. Although the selected metabolites were altered in the cerebellum, our enrichment analysis may suggest that these effects can also be present in the systemic circulation. For example, enrichment analysis data showed that khat exposure has strong interactions with drugs acting predominantly on proximal tubules of the kidney, SLC22A12 protein, aspirin, lactate-phenylalanine pathway, and natural killer cells. The findings in the present study indicate that repeated khat exposure is not only associated with psychoactive effects but also modulates the metabolomic profile in the cerebellum, which may further affect the pharmacokinetic profiles of various drugs, possibly due to cytochrome P 450 system interactions.

## 5. Conclusions

The present study found two novel findings, i.e., that repeated exposure of mice to khat is associated with altered levels in the cerebellum of various metabolites which are involved in the pathogenesis of different diseases, signaling pathways, and interactions with the pharmacokinetic profiles of other therapeutic drugs. Secondly, treatment with clavulanic acid in khat-exposed mice positively modified the metabolomic profiles in the cerebellum. In comparison to previous studies, our data showed the metabolomics and altered signaling pathways in the cerebellum part of the brain and the reversal of all of these changes by treatment with clavulanic acid. Our work suggests that beta lactam containing compounds not only attenuate drug seeking behavior but can also attenuate the altered metabolomic profiles in the cerebellum of animal models of substance-use disorders. The strength of our study was its evaluation of the effects of a herbal plant which is abused as psychoactive agent; we evaluated its impact on different metabolites in the cerebellum. This study may therefore be a source of information for local and global communities. Future studies are warranted to further explore the effects of khat on other metabolites in the cerebellum. In addition, a follow-up study should investigate the association between altered cerebellum metabolite levels and changes in proteins and neurotransmitters following exposure to khat.

## Data Availability

All data of the is shown in the manuscript.
